# Endoscopic single-scope placement of transendoscopic enteral tubing using a novel traction-assisted method in a child with autism spectrum disorder and refractory constipation

**DOI:** 10.1055/a-2724-7472

**Published:** 2025-12-15

**Authors:** Laihe Li, Yingchun Zhang, Ling Dong, Chongju Bao, Qing Cheng, Guili Xia

**Affiliations:** 1 559569 Shenzhen Hospital of Southern Medical University, Shenzhen, China

A 4-year to 11-month-old boy with autism spectrum disorder (ASD) and refractory constipation was scheduled for fecal microbiota transplantation (FMT). Colonoscopy revealed poor bowel preparation (Boston score: 4), tortuous and narrow lumen, and difficult scope advancement. To overcome this, we utilized a novel traction-assisted technique for one-step placement of transendoscopic enteral tubing (TET).

One end of a surgical suture was fixed to the TET tip, and the suture was externalized through the biopsy channel of a transparent cap-fitted colonoscope ( Fig. 1 ). This allowed the TET tip to align with the scope tip while preserving suction. The colonoscope with the TET attached was advanced into the colon. Gentle traction was applied via the suture to maintain alignment until the cecum was reached. The TET coils were then fixed to the colonic wall using metal clips ( Fig. 2 , Fig. 3 ). The final placement of the TET tube with the retained T-tube and surgical suture is shown in Fig. 4 . The entire procedure was performed in a single intubation, and the TET was successfully retained for 3 days without adverse events ( Video 1 ).

**Fig. 1:**
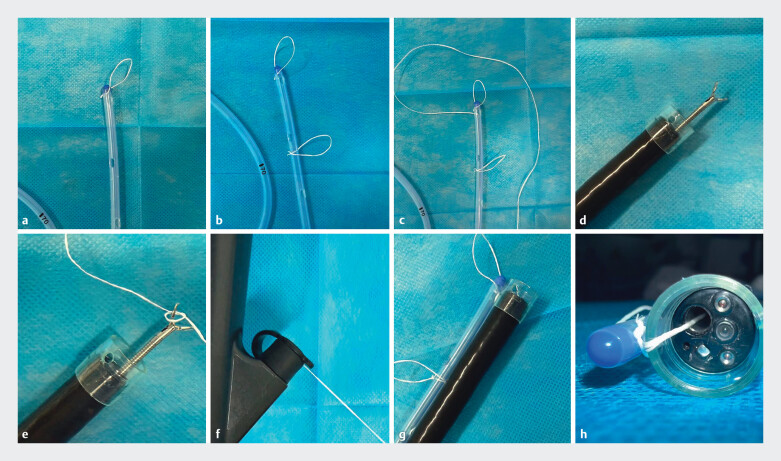
Preparation, assembly, and pre-insertion steps of the TET tube. **a, b** construction of the TET tube coils; **c** preparation and fixation of the traction line; **d, e** pulling of the traction line using the transparent cap and metal clip; **f** fixation of the tail end of the traction line; **g** alignment of the TET tube with the transparent cap; **h** occupation of the biopsy channel by the traction line.

**Fig. 2:**
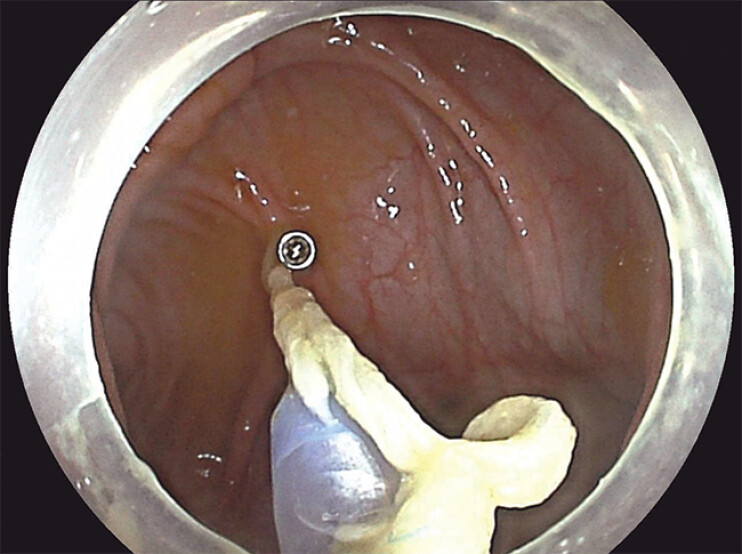
Endoscopic view showing fixation of the first loop of the TET tube with a metal clip.

**Fig. 3:**
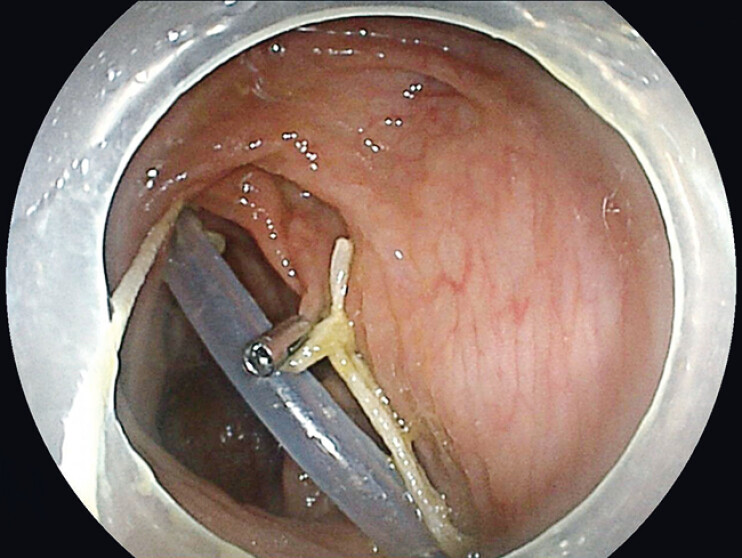
Endoscopic view showing fixation of the second loop of the TET tube with an additional metal clip.

**Fig. 4:**
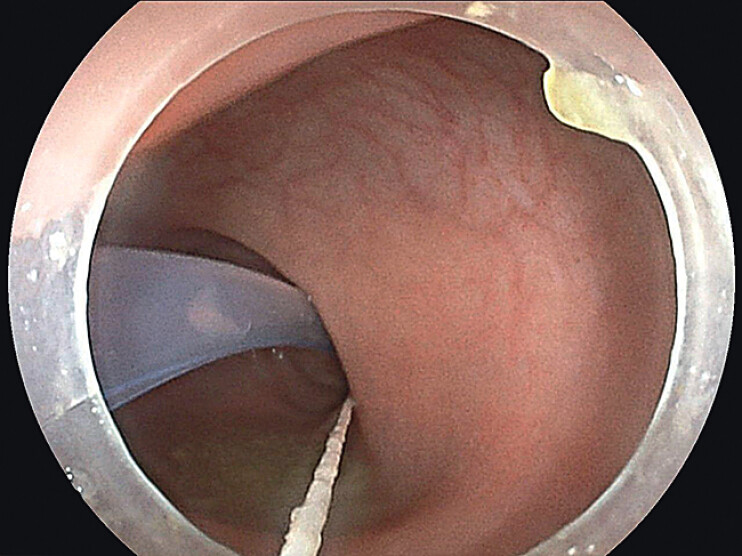
Endoscopic view showing the final placement of the TET tube with a retained T-tube and surgical suture, followed by withdrawal of the endoscope.

Single-scope placement of a TET tube using a novel traction-assisted method in a child with autism spectrum disorder.Video 1

This traction-assisted method enables efficient and secure one-step TET placement under colonoscopy, avoiding repeat intubation, reducing accessory costs, and minimizing patient burden.

Endoscopy_UCTN_Code_TTT_1AO_2AK

## Notes

Endoscopy E-Videos https://eref.thieme.de/e-videos
*E-Videos* is an open access online section of the journal *Endoscopy* , reporting on interesting cases and new techniques in gastroenterological endoscopy. All papers include a high-quality video and are published with a Creative Commons CC-BY license. Endoscopy E-Videos qualify for HINARI discounts and waivers and eligibility is automatically checked during the submission process. We grant 100% waivers to articles whose corresponding authors are based in Group A countries and 50% waivers to those who are based in Group B countries as classified by Research4Life (see: https://www.research4life.org/access/eligibility/ ). This section has its own submission website at https://mc.manuscriptcentral.com/e-videos .

